# Vesico-Vaginal Fistula, Cured by an Operation

**Published:** 1851-12

**Authors:** 


*Vesico-Vaginal Fistula, cured by an Operation.* By Prof. PANCOAST.—The October number of the Medical Examiner, contains a report of an exceedingly interesting operation performed by Professor Pancoast. The subject was a lady who had been delivered, in 1848, of a child by mutilating instruments. After her recovery from her fatigue and exhaustion, she discovered that she had constant *stillicidium urinæ*, with all its attendant afflictions. The cause of the distress "depended upon a laceration affecting the urethra, so nigh to the neck of the bladder as to prevent the *sphincter vesicæ* from controlling the escape of the urine."

Professor Pancoast found it difficult, after various efforts, "to bring the injured part within the reach of his instruments, and he urged the patient to postpone the operation some months. In order to make the case more tractable for an operation, Professor Meigs prepared a peculiarly shaped canula, with a small funnel adapted to one end of a spindle, and a perforated disc at the other end. Professor Meigs, who reports the case, says:

"Such an instrument placed in the urethra, would not be thrust forth except the patient should make violent efforts to expel it. We hoped that by thus preventing the continual flow of the urine through the lacerated part, it might tend to close up so much as to render an operation practicable and successful; and I explained to her that such a conductor for the urine, if it could be worn for a long time, would perhaps serve at last as a cure, without resort to operation by the knife. She was begged to persist in the trial for several months — but came back again in about 150 days — being impatient of her sufferings.

"We thought great good was effected by the use of the canula, which she used during the greater part of the time, being, however, during her absence from town, annoyed to that degree, as to be sometimes obliged to suspend the use of it — which was reasonably to be expected.

"On a re-examination of the fistula in connection with Dr. Pancoast, on the 13th of August, we found it much more easily reached with instruments than on the former visit of the patient, and the performance of the operation for its cure was determined on. On the 16th day of August, Dr. P., assisted by Mr. C. Neff, and by myself, the patient being on her knees upon a bed, proceeded to the operation. The callous margin of the fistula was first pared away — the mucous membrane on the right side of the wound was then seized by Dr. P. with a pair of delicate hooked forceps, and detached with scissors curved on the flat, for the space of five-eighths of an inch around the semi-circumference of the orifice. The next stage of the operation was to obtain a corresponding surface on the opposite or further side, to overleap that already made. This was gained by Dr. P. in the following mode. The right lip of the orifice was grasped superficially with the forceps, and split midway between the two mucous membranes, to the depth of half an inch, with a lance-shaped knife curved on the flat, making, as it were, two layers of the vesico-vaginal septum, for the space of nearly an inch in length and half an inch in depth."

Professor Meigs pays a well-deserved compliment to Dr. Sims, of Montgomery, Ala., in connection with this operation. The complimentary remarks contain an explanation of the suggestion made by Dr. Sims, and for that reason they are quoted at length. Professor Meigs says:

"In a conversation with him in the end of July, 1851, he informed me of his many successful operations in vesico-vaginal fistula — a success which, in my humble opinion, entitles him to the praise and gratitude of our whole profession, which is always advanced by the ingenious and rational improvements made by such individuals, and which constitute a real progress of the art. Dr. Sims communicated to me the fact, that if the patient, laboring under this accident, be placed in a certain position, the injured parts may be readily brought, not only in sight, but so exposed as to render them easily accessible to the knife, the scissors, &c., &c.

"His own judgment prompted the discovery of this method, which consists in placing the patient on her knees with the ossa femoris standing in a vertical position, while the summit of the thorax touches the table or bed on which she is placed.

"If such a posture be maintained for a few minutes, the fall of the abdominal and pelvic viscera downward toward the diaphragm, allows the os externum to relax, to open, and at last to offer so wide an aperture, that the cervix uteri may be clearly seen as in a metroscope. In the meantime, the fall of the viscera continuing, the walls of the vagina, to avoid the tendency to vacuum caused by the retreat of the pelvic contents toward the diaphragm, tend to expand, and like a balloon leave a spherical or orbicular cavity, as if the vagina was filled with a child's head, whereas it is filled with air.

"This discovery is in the highest degree applicable to the uses of curative surgery in vesico-vaginal fistula.

"The great experience and success of Dr. Sims will enable him, it is hoped, soon to give his results to the profession. In the meantime, I trust I shall give no offence while in describing the several steps of the operation on my patient, if I take this early occasion to thank Dr. Sims for his information, and to acknowledge how much I am his debtor therefor.

"From the moment of completing the operation above described, not a drop of urine escaped through the fissure. She wore the spindle-shaped canula during the ten or twelve days following, and then removed it. The ligatures were easily cut away and picked out with dressing forceps, and we had the happiness to see the patient completely relieved of the effects of an accident, which nothing but a religious resignation could have enabled her to bear with patience, or to regard life otherwise than as a distressing burden, to be borne only because it was God's will that she should patiently endure its weight. The cure was rapid and complete."—*From Dr. Bell's excellent Summary of Prac. Med. in Western Med. and Surg. Journal.*

